# A Case Report of Hantavirus Capillary Leak Syndrome in the Southwestern United States

**DOI:** 10.1155/crdi/9919759

**Published:** 2026-10-28

**Authors:** Devika Sindu, Raed Suyyagh, Abdalla Fadda, Mathew Fain, Josna Padiyar

**Affiliations:** ^1^ School of Medicine Creighton University Phoenix Arizona USA creighton.edu; ^2^ Norton Thoracic Institute St. Joseph’s Hospital and Medical Center Phoenix Arizona USA dignityhealth.org

**Keywords:** ARDS, capillary leak, case report, hantavirus cardiopulmonary syndrome (HCPS), pulmonary edema, shock

## Abstract

Hantavirus cardiopulmonary syndrome (HCPS) is a rare but frequently fatal viral disease endemic to the Americas, transmitted primarily through inhalation of aerosolized rodent excreta. Early symptoms are nonspecific, but the disease can rapidly progress to severe respiratory compromise and shock due to profound capillary leak. We report the case of a previously healthy 20‐year‐old man who initially presented with abdominal pain, nausea, vomiting, diarrhea, and progressive weakness after occupational exposure on a ranch in southwestern Arizona. He quickly developed hemoconcentration (hematocrit 58%), thrombocytopenia (17,000 cells/μL), septic shock, and noncardiogenic pulmonary edema. The initial infectious workup was negative. His clinical course was complicated by respiratory failure requiring intubation and vasopressor support. Computed tomography of his chest showed pulmonary edema and large bilateral pleural effusions despite aggressive diuresis, requiring bilateral chest tubes. He gradually improved with supportive care, was successfully extubated, weaned off oxygen, and discharged home with outpatient rehabilitation. His diagnosis was later confirmed with a serum ELISA test which was positive for hantavirus IgM and IgG. This case highlights the diagnostic complexity of HCPS, particularly when gastrointestinal symptoms predominate early in the illness. In endemic areas with rodent exposure history, hemoconcentration and thrombocytopenia, along with signs of volume overload and rapidly progressive acute respiratory failure, should raise suspicion for hantavirus infection. Identification and supportive care are the mainstay of treatment. Although this patient did not require ECMO, transfer to an ECMO center may be beneficial to support recovery during the cardiopulmonary phase, as recommended by the Centers for Disease Control.

## 1. Introduction

Hantaviruses are enveloped single‐stranded RNA viruses that can cause zoonotic diseases in humans [1], including the two most common, hemorrhagic fever with renal syndrome (HFRS) and hantavirus cardiopulmonary syndrome (HCPS) [2]. HFRS is caused by Old World orthohantaviruses and is seen in Asia and Europe [3]. Whereas, HCPS was first identified in the southwestern United States in the early 1990s and is caused by New World hantaviruses [4]. The primary route of transmission is inhalation of aerosolized particles from the urine, feces, or saliva of infected rodents. Although HCPS is rare with an annual incidence of 0.04 to 0.19 cases/million persons [4], it is associated with a high mortality rate ranging from 30% to 50% and poses a diagnostic challenge due to its nonspecific prodromal symptoms [5]. The disease typically progresses from a febrile phase to a rapidly deteriorating cardiopulmonary phase characterized by noncardiogenic pulmonary edema, hypotension, and shock resulting from widespread capillary leak [5]. Clinical diagnosis often relies on a combination of epidemiological context, laboratory abnormalities such as thrombocytopenia and elevated hematocrit, and a high index of suspicion, particularly in endemic regions [6, 7]. We present a case of severe HCPS in a previously healthy young man who developed profound capillary leak syndrome with respiratory failure and shock. This case emphasizes the diagnostic importance of gastrointestinal‐predominant prodromal symptoms, early risk stratification, and timely escalation of supportive care. Per institutional policy, this single‐patient case report did not require formal Institutional Review Board approval. No written consent has been obtained from the patients as there is no patient identifiable data included in this case report.

## 2. Case Presentation

A 20‐year‐old man with no known medical history presented to an outside hospital with over six days of abdominal pain, nausea, vomiting, diarrhea, and generalized weakness. He had recently returned from a vacation in Mexico, where he was evaluated at an urgent care center for similar symptoms and was treated symptomatically with intravenous fluids and antiemetics. Upon his return to southwestern Arizona, where he worked on a ranch, he sought further evaluation in the emergency department due to persistent symptoms. He was febrile but hemodynamically stable on presentation. Laboratory results were notable for leukocytosis with neutrophilia (white cell count 38,100 cells/μL), bandemia (10.4%), hemoconcentration (hemoglobin 20.3 g/dL, hematocrit 58%), thrombocytopenia (17,000 cells/μL), lactic acidosis (4.8 mmol/L), and elevated procalcitonin (2.43 ng/mL). Chest CT (Figure 1A) revealed patchy parenchymal opacities suggestive of pulmonary edema, predominant right middle lobe opacities, which may suggest superimposed infection/aspiration, and small bilateral pleural effusions. Subsequently, he became progressively hypoxic and hypotensive, requiring mechanical ventilation and vasopressor support. Patient samples were pan‐cultured, and broad‐spectrum antibiotics were started.

**FIGURE 1:**
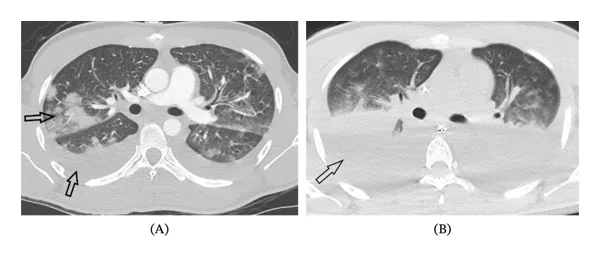
Chest CT on presentation (A) showing patchy parenchymal opacities suggestive of pulmonary edema, predominant right middle lobe opacities which may suggest superimposed infection/aspiration, and small bilateral pleural effusions; and three days later (B) showing worsening pulmonary edema and large bilateral pleural effusions suggestive of capillary leak, leading to the placement of bilateral chest tubes.

The patient was subsequently transferred to our center for a higher level of care and evaluation for extracorporeal membrane oxygenation (ECMO). He was maintained on lung‐protective ventilation (tidal volume 5.6 mL/kg, PEEP 12, and FiO2 70%–100%). Empiric antibiotics (piperacillin‐tazobactam, vancomycin, and doxycycline), stress‐dose steroids, and vasopressor support (norepinephrine 11 mcg/min, vasopressin 0.03 U/min) were continued. A transthoracic echocardiogram showed normal left and right heart function with a small pericardial effusion. Bronchoalveolar lavage cultures showed no microbial growth. Respiratory viral panels, bacterial cultures, and fungal serologies were negative. Bone marrow biopsy was negative for hematologic malignancies. On Day 3, a repeat chest CT showed worsening pulmonary edema, patchy peribronchovascular ground‐glass opacities that may represent alveolar edema, interlobular septal thickening, and large bilateral pleural effusions with adjacent atelectasis (Figure 1B); diuretics were started to maintain a net negative fluid balance (at least–1L every day). Bilateral chest tubes were placed, draining 1L of serous fluid each, and pleural fluid studies revealed a cell count of 129 (45% neutrophils, 12% lymphocytes, 30% macrophages), a pleural fluid protein level of 2.5 g/dL (serum protein 5.3 g/dL), and a pleural fluid lactate dehydrogenase (LDH) of 301 U/L (serum LDH 648 U/L, upper limit of normal 243 U/L). His B‐type natriuretic peptide was elevated (527.8 pg/mL). Abdominal CT also showed signs of volume overload with small‐volume ascites, pelvic free fluid, mesenteric edema, and diffuse anasarca; diuresis continued. He was gradually weaned off vasopressors, and ventilator parameters including FiO2 and PEEP gradually decreased. He was successfully extubated to a high‐flow nasal cannula on Day 5, and antibiotics were discontinued. His clinical status and laboratory abnormalities gradually improved (Figures 2 and 3), and his right and left chest tubes were removed on Days 8 and 9, respectively. The patient was net negative at 10.9 L during his inpatient stay. Additionally, antinuclear, antiglomerular basement membrane, myeloperoxidase, and serine protease‐3 antibodies were negative. He was discharged home 10 days after presentation with outpatient follow‐up. Eventually, ELISA testing for hantavirus IgM and IgG returned positive from a reference laboratory about 10 days after presentation, confirming the diagnosis of HCPS.

**FIGURE 2:**
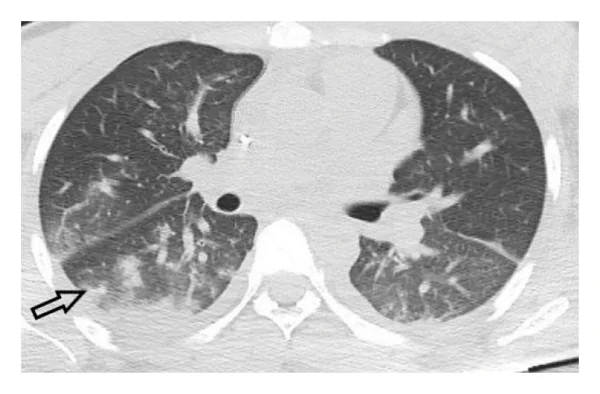
Chest CT two days after bilateral chest tube insertion and diuresis showed improvement with scattered ground‐glass opacities and decreased small bilateral pleural effusions.

**FIGURE 3:**
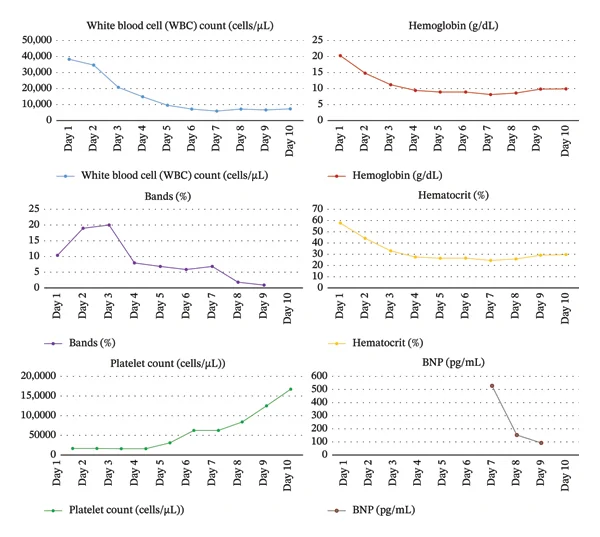
Trends in laboratory results during the patient’s hospital stay reflect the transition from cardiopulmonary phase to convalescence. The patient was extubated and vasopressors were stopped on day 5. Right and left chest tubes were removed on day 8 and 9, respectively.

## 3. Discussion

Since the initial recognition of HCPS during the 1993 Four Corners outbreak, approximately 30 cases have been reported annually in the United States [4, 8]. HCPS most commonly affects previously healthy young adults with occupational or environmental rodent exposure. Over 20 strains of hantaviruses can cause HCPS, but the primary culprits are Sin Nombre virus and Andes virus in North and South America, respectively [3]. Rodents excrete hantaviruses in saliva, urine, and feces, and humans are infected when inhaling the secreted viruses or rarely by rodent bites [2]. This case illustrates a classical but diagnostically challenging presentation of HCPS in a young ranch worker with substantial exposure to rodent‐contaminated environments.

### 3.1. Gastrointestinal‐Predominant Prodrome as a Diagnostic Pitfall

The incubation period of hantavirus ranges from 1 to 3 weeks. The prodromal phase of HCPS frequently resembles a nonspecific viral or gastrointestinal illness and may include abdominal pain, nausea, vomiting, and diarrhea before overt respiratory symptoms develop. Gastrointestinal‐predominant presentations may delay diagnosis because clinicians often initially prioritize gastroenteritis, abdominal sepsis, arboviral disease, or other infectious syndromes. In this case, several bedside “red flags” increased suspicion for HCPS despite the nonspecific prodrome: severe thrombocytopenia, marked hemoconcentration, granulocytic left shift, rapid respiratory deterioration, and rodent exposure in an endemic region.

Clinicians at the University of New Mexico developed five criteria to guide clinical decisions and make a presumptive diagnosis during this phase: thrombocytopenia, hemoconcentration, granulocytic left shift, absence of toxic changes, and more than 10% immunoblasts. Blood smears meeting four of five criteria had high sensitivity (96%), specificity (99%), and positive predictive value (83%) for infection [6, 7]. Notably, our patient met the criteria, and confirmatory testing for hantavirus was dispatched at this stage.

### 3.2. Differential Diagnosis and Early Clinical Decision‐Making

This case highlights two critical clinical decision‐making windows. During the initial prodromal phase, differential considerations included viral gastroenteritis, leptospirosis, dengue/arboviral infections, bacterial sepsis, rickettsioses, influenza‐like illness, Epstein–Barr virus, human bocavirus, hematologic malignancies, and abdominal sources of infection. As the patient progressed to respiratory failure and shock, the differential expanded to include severe sepsis‐associated ARDS, vasculitis with diffuse alveolar injury or pulmonary hemorrhage syndromes (e.g., granulomatosis with polyangiitis and Goodpasture syndrome), Aspergillus, Pneumocystis, Legionella, Chlamydia, Mycoplasma, Q fever, and HCPS.

Several features substantially increased the likelihood of HCPS: rapid onset pulmonary edema with preserved cardiac function, thrombocytopenia, marked hemoconcentration, diffuse capillary leak, and epidemiologic exposure to rodent‐contaminated ranch environments. The broad diagnostic evaluation was appropriate because early HCPS frequently mimics other severe infectious and inflammatory syndromes.

### 3.3. Pathophysiology of Capillary Leak and Pulmonary Edema

Pathogenesis of hantavirus infection is characterized by increased vascular permeability, as the virus principally targets endothelial cells of pulmonary and renal capillaries [9]. It is hypothesized that alpha and beta‐3 integrins act as hantavirus receptors on endothelial cells, and their inactivation contributes to the deregulation of vascular endothelial growth factor, leading to altered expression and internalization of vascular endothelial cadherins, damaging the endothelial barrier [10–12]. In HCPS, this accounts for the capillary leak syndrome by pulmonary edema, pleural effusions, hemoconcentration, hypotension, ascites, mesenteric edema, and anasarca, all of which were present in this patient. Thrombocytopenia is a common and early marker of disease severity and is thought to result from increased endothelial activation and consumption.

Although echocardiography demonstrated preserved ventricular systolic function, BNP elevation raised consideration of transient myocardial involvement, which has been described in HCPS. Therefore, pulmonary edema in HCPS may reflect a mixed process involving both severe endothelial leak and variable myocardial depression. In this case, preserved ventricular function and the overall hemodynamic pattern favored predominantly noncardiogenic pulmonary edema.

### 3.4. Risk Stratification and Escalation of Care

This patient presented with several features associated with severe HCPS, including profound thrombocytopenia, hemoconcentration, vasopressor‐dependent shock, respiratory failure requiring mechanical ventilation, diffuse pulmonary edema, and elevated lactate levels. These high‐risk features justified early transfer to an ECMO center and close hemodynamic monitoring.

Despite his severe presentation, the patient experienced favorable recovery without ECMO support. Potential explanations include early recognition of capillary leak physiology, prompt intensive care support, lung‐protective ventilation, vasopressor therapy, conservative fluid management, patient age, gender, and the absence of significant medical comorbidities. Recent systematic review data emphasize the importance of early referral to an ECMO center for advanced supportive therapies in severe HCPS [13].

### 3.5. Current and Emerging Therapies

Management of HCPS remains largely supportive. Mechanical ventilation, hemodynamic support, conservative fluid management, and early recognition of capillary leak physiology are central to care. Venoarterial ECMO may be used in cases with cardiac index < 2.5 L/minute/m^2^ despite pressor and inotropic support [14]. Small placebo‐controlled studies using ribavirin [15] or high‐dose methylprednisolone [16] did not show clear benefits. A nonrandomized pre‐ and postintervention study (ISRCTN72088243) to assess the efficacy of tocilizumab in reducing mortality in HCPS is underway in Argentina [17]. A recent case‐series based on this trial seemed to show some promise for IL‐6 inhibition in four of the five HCPS patients who survived to ICU discharge after treatment with tocilizumab [18]. However, given the high mortality and a lack of established treatment options, prevention of hantavirus infection by implementing measures to limit contact with rodents is of utmost importance.

### 3.6. Epidemiology and Long‐Term Outcomes

Arizona and the broader Four Corners region remain endemic areas for Sin Nombre virus transmission because of persistent deer mouse reservoirs and environmental exposure risks in rural settings. Occupational exposure involving barns, feed storage areas, sweeping of rodent‐contaminated spaces, and aerosolization of rodent excreta remain important risk factors for infection.

Long‐term outcomes among HCPS survivors remain incompletely characterized. Although many patients recover functional independence, persistent fatigue, exertional intolerance, and psychological distress have been reported after severe disease. In this case, the patient demonstrated favorable short‐term recovery and was able to return to routine activities within weeks of discharge, although mild exertional fatigue persisted at follow‐up.

## 4. Conclusion

The strength of this case report lies in serologic confirmation of Hantavirus using ELISA and elaborate clinical details on its presentation, complications, and management. Although it is limited to a single patient, it underscores the diagnostic complexity and clinical severity of HCPS. Notably, gastrointestinal‐predominant prodromal symptoms may obscure early recognition and delay diagnosis. Although rare, HCPS should remain high on the differential for patients presenting with hemoconcentration, thrombocytopenia, rapid‐onset respiratory failure, and shock, especially in endemic regions with known rodent exposure. Early risk stratification, aggressive supportive management, and timely referral to centers capable of advanced cardiopulmonary support are essential to optimize outcomes.

## Author Contributions

Devika Sindu, Raed Suyyagh, Abdalla Fadda, Mathew Fain, and Josna Padiyar made significant contributions to the conception or design of the work, acquisition of data, drafting the work, and revising it critically for important intellectual content, in accordance with ICMJE guidelines. All authors agree to be accountable for all aspects of the work in ensuring that questions related to the accuracy or integrity of any part of the work are appropriately investigated and resolved.

## Funding

This research did not receive any specific grant from funding agencies in the public, commercial, or not‐for‐profit sectors.

## Disclosure

The authors have approved the final version to be published.

## Conflicts of Interest

The authors declare no conflicts of interest.

## Supporting Information

Additional supporting information can be found online in the Supporting Information section.

## Supporting information


**Supporting Information** Hantavirus Case Study_CARE‐checklist‐English‐2013.

## Data Availability

The authors have nothing to report.
