# Supplementary material for: A Case Report of Hantavirus Capillary Leak Syndrome in the Southwestern United States
Source: Case Rep Infect Dis. 2026 Oct 28;2026:9919759. doi: 10.1155/crdi/9919759 (PMC13715490; doi:10.1155/crdi/9919759)
Supplement: Supplementary file 1 [file CRDI-2026-9919759-s001.pdf]

CARE Checklist of information to include when writing a case report

CARE case report guidelines

(cc) BY-NC-ND

Topic	Item	Checklist item description	Reported on Line
Title	1	The diagnosis or intervention of primary focus followed by the words "case report"	1. Line 1
Key Words	2	2 to 5 key words that identify diagnoses or interventions in this case report, including "case report"	2. line 16
Abstract (no references)	3a	Introduction: What is unique about this case and what does it add to the scientific literature?	3a. lines 37-39
	3b	Main symptoms and/or important clinical findings	3b. lines 28-31
	3c	The main diagnoses, therapeutic interventions, and outcomes	3c. lines 36-37
	3d	Conclusion—What is the main "take-away" lesson(s) from this case?	3d. lines 37-44
Introduction	4	One or two paragraphs summarizing why this case is unique (**may include references**)	4. lines 59-64
Patient Information	5a	De-identified patient specific information.	5a. line 66
	5b	Primary concerns and symptoms of the patient.	5b. lines 66-72
	5c	Medical, family, and psycho-social history including relevant genetic information	5c. lines 70-71 (endemic location)
	5d	Relevant past interventions with outcomes	5d. lines 68-80
Clinical Findings	6	Describe significant physical examination (PE) and important clinical findings.	6. lines 81-108
Timeline	7	Historical and current information from this episode of care organized as a timeline	7. Throughout case history, lines 66-108
Diagnostic Assessment	8a	Diagnostic testing (such as PE, laboratory testing, imaging, surveys).	8a. lines 66-108
	8b	Diagnostic challenges (such as access to testing, financial, or cultural)	8b. lines 119-133
	8c	Diagnosis (including other diagnoses considered)	8c. lines 135-147
	8d	Prognosis (such as staging in oncology) where applicable	8d. lines 166-176
Therapeutic Intervention	9a	Types of therapeutic intervention (such as pharmacologic, surgical, preventive, self-care)	9a. Throughout case history, lines 66-108
	9b	Administration of therapeutic intervention (such as dosage, strength, duration)	9b. Throughout case history, lines 66-108
	9c	Changes in therapeutic intervention (with rationale)	9c. Throughout case history, lines 66-108
Follow-up and Outcomes	10a	Clinician and patient-assessed outcomes (if available)	10a. 197-199
	10b	Important follow-up diagnostic and other test results	10b. 106-108
	10c	Intervention adherence and tolerability (How was this assessed?)	10c. 105-106; 197-199
	10d	Adverse and unanticipated events	10d. Throughout case history, lines 66-108
Discussion	11a	A scientific discussion of the strengths AND limitations associated with this case report	11a. lines 201-204
	11b	Discussion of the relevant medical literature **with references**.	11b. lines 110-199
	11c	The scientific rationale for any conclusions (including assessment of possible causes)	11c. lines 106-108
	11d	The primary "take-away" lessons of this case report (without references) in a one paragraph conclusion	11d. lines 201-207
Patient Perspective	12	The patient should share their perspective in one to two paragraphs on the treatment(s) they received	12. Not available, patient lost to follow-up.
Informed Consent	13	Did the patient give informed consent? Please provide if requested	13. Not required by IRB
